# Supplementary material for: A d10-Cd Cluster Containing Sandwich-Type Arsenotungstate Exhibiting Fluorescent Recognition of Carcinogenic Dye in Methanol
Source: Molecules. 2024 Nov 2;29(21):5193. doi: 10.3390/molecules29215193 (PMC11547473; doi:10.3390/molecules29215193)
Supplement: Supplementary file 1 [file molecules-29-05193-s001.zip › molecules-3261569-supplementary.pdf]

Supplementary Information

A d^{10}-Cd Cluster Containing Sandwich-Type Arsenotungstate Exhibiting Fluorescent Recognition of Carcinogenic Dye in Methanol

Feng Wang [1,†], Xiang Ma [2,*,†], Haodong Li [1], Ziqi Zhao [2], Lele Zhang [1], Yutong Zhao [1], Haipeng Su [2], Zeqi Wang [1], Changchun Li [2] and Jiai Hua [1,*]

1 Chemistry and Chemical Engineering Department, Taiyuan Institute of Technology, Taiyuan 030008, China; wangf@tit.edu.cn (F.W.); 16603265568@163.com (H.L.); 16613802856@163.com (L.Z.); 19581525302@163.com (Y.Z.); wzq15003489249@163.com (Z.W.)

2 Laboratory of Biochemistry and Pharmacy, Taiyuan Institute of Technology, Taiyuan 030008, China; 13394412558@163.com (Z.Z.); 19935347213@163.com (H.S.); l17850742078@163.com (C.L.)

* Correspondence: maxiang@tit.edu.cn (X.M.); huaja@tit.edu.cn (J.H.); Tel.: +86-351-3569476 (J.H.)

† These authors contributed equally to this work.

Table S1 Crystallographic data and structural refinements for compound I

Empirical formula	$C_{18}H_{86}As_2Cd_4N_{12}Cl_2O_{75}W_{18}$
Formula weight	5650.62
Crystal system	Triclinic
Space group	*P*–*1*
a / Å	12.5169(15)
b / Å	12.5282(16)
c / Å	15.952(3)
α / deg	99.623(3)
β / deg	98.647(3)
γ / deg	112.828(2)
V / Å^3	2208.5(6)
Z	1
D_c / g cm^{-3}	4.249
μ / mm^{-1}	25.187
T / K	293(2)
Limiting indices	$-14 \leq h \leq 14$
	$-14\leq k \leq 14$
	$-12 \leq l \leq 18$
Measured reflections	10995
Independent reflections	7649
R_{int}	0.0939

Data / restrains / parameters	7649/ 262 /634
GOF on F^2	0.996
Final R indices [$I > 2\sigma(I)$]	R_1 = 0.0785, wR_2 = 0.1726
R indices (all data)	R_1 = 0.0933 wR_2 = 0.1844

Table S2 Selected bond length (Å) for compound I

W(1)-O(1)	1.747	W(2)-O(2)	1.754
W(1)-O(10)	1.774	W(2)-O(11)	1.773
W(1)-O(12)	1.924	W(2)-O(13)	1.943
W(1)-O(30)	1.963	W(2)-O(12)	1.941
W(1)-O(25)	2.020	W(2)-O(14)	1.999
W(1)-O(31)	2.322	W(2)-O(31)	2.360
W(3)-O(3)	1.715	W(4)-O(4)	1.711
W(3)-O(25)	1.859	W(4)-O(20)	1.767
W(3)-O(14)	1.884	W(4)-O(13)	1.887
W(3)-O(19)	1.935	W(4)-O(17)	1.997
W(3)-O(15)	1.937	W(4)-O(18)	2.002
W(3)-O(31)	2.483	W(4)-O(32)	2.355
W(5)-O(5)	1.710	W(6)-O(6)	1.728
W(5)-O(18)	1.881	W(6)-O(22)	1.786
W(5)-O(21)	1.888	W(6)-O(17)	1.873
W(5)-O(15)	1.895	W(6)-O(28)	1.989
W(5)-O(16)	1.959	W(6)-O(21)	1.995
W(8)-O(8)	1.718	W(6)-O(32)	2.369
W(8)-O(26)	1.805	W(7)-O(7)	1.711
W(8)-O(29)	1.883	W(7)-O(27)	1.849
W(8)-O(28)	1.900	W(7)-O(19)	1.898
W(8)-O(23)	1.981	W(7)-O(16)	1.906
W(8)-O(33)	2.332	W(7)-O(23)	1.908
W(9)-O(9)	1.715	Cd(1)-O(11)	2.221
W(9)-O(24)	1.780	Cd(1)-O(10)	2.243
W(9)-O(30)	1.889	Cd(1)-O(26)#1	2.286
W(9)-O(29)	1.969	Cd(1)-O(22)#1	2.315
W(9)-O(27)	2.051	Cd(1)-Cl(1)	2.512
W(9)-O(33)	2.378	Cd(1)-O(34)	2.510
Cd(2)-O(24)	2.165	Cd(2)-O(34)	2.340
Cd(2)-O(20)#1	2.175	As(1)-O(34)	1.660
Cd(2)-O(22)#1	2.254	As(1)-O(32)	1.673
Cd(2)-O(26)	2.290	As(1)-O(33)	1.690

Cd(2)-O(34)#1	2.339	As(1)-O(31)	1.708
